# The impact of maternal and early life malnutrition on health: a diet-microbe perspective

**DOI:** 10.1186/s12916-020-01584-z

**Published:** 2020-05-12

**Authors:** Andrew J. Forgie, Kelsea M. Drall, Stephane L. Bourque, Catherine J. Field, Anita L. Kozyrskyj, Benjamin P. Willing

**Affiliations:** 1 Department of Agricultural, Food and Nutritional Science, University of Alberta, Edmonton, Alberta Canada; 2 Department of Pediatrics, University of Alberta, Edmonton, Alberta Canada; 3 Department of Anesthesiology & Pain Medicine, University of Alberta, Edmonton, Alberta Canada

**Keywords:** Undernutrition, Malnutrition, Diet, Microbiome, Gastrointestinal, Disease, Health

## Abstract

**Background:**

Early-life malnutrition may have long-lasting effects on microbe-host interactions that affect health and disease susceptibility later in life. Diet quality and quantity in conjunction with toxin and pathogen exposure are key contributors to microbe-host physiology and malnutrition. Consequently, it is important to consider both diet- and microbe-induced pathologies as well as their interactions underlying malnutrition.

**Main Body:**

Gastrointestinal immunity and digestive function are vital to maintain a symbiotic relationship between the host and microbiota. Childhood malnutrition can be impacted by numerous factors including gestational malnutrition, early life antibiotic use, psychological stress, food allergy, hygiene, and exposure to other chemicals and pollutants. These factors can contribute to reoccurring environmental enteropathy, a condition characterized by the expansion of commensal pathobionts and environmental pathogens. Reoccurring intestinal dysfunction, particularly during the critical window of development, may be a consequence of diet-microbe interactions and may lead to life-long immune and metabolic programming and increased disease risk. We provide an overview of the some key factors implicated in the progression of malnutrition (protein, fat, carbohydrate, iron, vitamin D, and vitamin B12) and discuss the microbiota during early life that may contribute health risk later in life.

**Conclusion:**

Identifying key microbe-host interactions, particularly those associated with diet and malnutrition requires well-controlled dietary studies. Furthering our understanding of diet-microbe-host interactions will help to provide better strategies during gestation and early life to promote health later in life.

## Background

Microbes of the gastrointestinal tract are associated to health and disease. It is well established that their functional coexistence within the gut can be manipulated by altering their environment, of which host nutrition has the greatest impact [1]. Although disease causality has not been directly attributed to the microbiota, researchers hypothesized that early-life exposure and assembly of microbes in the gut influences host development [2, 3]. The developmental origins of health and disease (DOHaD) hypothesis proposes that an early-life window exists where environmental exposures, including the mode of birth, nutrition, breastfeeding, infection, and antibiotics, lead to programming effects that can affect long-term health [4]. During the first 1000 days of life, host immune, endocrine, metabolic, and other developmental pathways mature in tandem with the microbiome to achieve a mutualistic relationship [5]. Even transient disturbances to microbial communities (“dysbiosis”) during this critical window of development have been associated with immune-mediated, metabolic, and neurodevelopmental disorders [6–8]. In this review, we discuss the potential implication of maternal and infant diet-induced microbial and host changes surrounding early-life protein, fat, carbohydrate, iron, vitamin D, and vitamin B12 malnutrition.

### Contribution of microbes in health and disease

Despite mounting evidence associating the microbiota to health and disease, their causal contribution remains poorly understood. Microbes are niche-specific, meaning they are adapted to a particular lifestyle or ecological niche [9]. The microbial signature, which reflects the presence and activity of microbes, changes in response to diverse exogenous factors, including diet, disease, antibiotic usage, and host genetics [10]. For this reason, distinct microbial signatures have been identified in various human diseases, including obesity, diabetes, colorectal cancer, irritable bowel disease (IBD), liver cirrhosis, and pulmonary tuberculosis [11–13]. Although microbial signatures are associated with disease, it is unclear whether they are causally linked or simply a result of altered gut ecology due to metabolic and immunological changes occurring in the diseased host. Despite the fact that microbial contributions may be secondary to the causal agent of the disease, research on environmental enteropathy (EE) strongly suggests their presence is required for disease pathogenesis and indicates that microbes play a key role.

EE, also known as environmental enteric dysfunction, is a major contributor to early childhood stunting [14]. EE is a subclinical disorder associated with altered diet-microbe-host interactions and characterized by intestinal injury, nutrient malabsorption, and inflammation [15, 16]. EE can induce stunting and wasting through poor digestion and autoimmune dysfunction rather than food shortage [14]. Brown and colleagues show in a mouse model that specific bacteria are required for the development of EE [17]. Researchers were able to recreate the full effects of human EE by gavaging a defined mixture of Bacteroidales species and *Escherichia coli* to mice fed a protein-deficient diet (7% of the diet). However, when given an isocaloric protein-sufficient diet (20% of the diet), mice that were colonized with the same bacterial mixture did not develop EE symptoms. This suggests that both diet and microbes are necessary to induce EE.

Observations in mouse models reflect what has been observed in clinical studies, where EE symptoms are associated with both a malnourished diet and specific microbial signatures [18–20]. Metabolomic and proteomic analysis of blood plasma combined with metagenomic analyses of fecal samples revealed distinct microbe and host functions between healthy children and those with severe acute malnutrition when consuming the same therapeutic diet. As the children transitioned from severe to moderate acute malnutrition, their proteomic profiles became more similar to that of healthy children [19]. A microbiota-direct dietary intervention to these children had a greater impact than conventional therapy to restore microbiota structure and health [20]. Developmentally, a microbiota-direct dietary intervention increased biomarkers and mediators of growth, bone formation, neurodevelopment, and immune function towards a healthy phenotype [19]. Gnotobiotic mice and piglet models colonized with malnourished Malawian microbiota and fed a low caloric nutrient-deficient diet, resulted in weight loss and metabolic profiles distinctive of EE [18]. Kau et al. were able to show that a higher proportion of *Enterobacteriaceae* members relative to *Akkermansia municiphila* and *Clostridium scindens* in malnourished Malawian children was indicative of a pathogenic community related to malnutrition [21]. Using mouse models, researchers confirmed that a combination of diet and *Enterobacteriaceae*, *Enterococcus*, and Bacteroidetes members are required for EE pathogenesis.

### Pathobionts

Gastrointestinal dysbiosis is characterized by the loss of beneficial commensal microbe-host interactions and expansion of some commensal organisms, known as pathobionts, that exert pro-inflammatory effects on the host [22]. Pathobionts are opportunistic bacteria that pose a unique challenge in malnutrition research because their pathogenicity is dictated by host diet and immune function [23]. Numerous microbes have been identified as major contributors of gut dysbiosis and inflammation linked to disease, including commensal *Escherichia coli* strains [24], *Helicobacter hepaticus* [25], *Bilophila wadsworthia* [26], *Bacteroides fragilis* [27], *Fusobacterium nucleatum* [28], *Enterococcus faecalis* [29], and *Akkermansia muciniphila* [30]. Although diet, antibiotic, infection, and intestinal inflammation are the primary triggers of dysbiosis, pathobionts can exacerbate gastrointestinal dysfunction [22]. The impact of pathobiont overgrowth in the gastrointestinal tract on health may also be context-dependent. For example, *Akkermansia muciniphila* has been shown to improve glucose control [31, 32], but has also been shown to exacerbate infections and colitis [33, 34]. Reoccurring EE may be a consequence of poor diet-microbe-host interactions that limit the capacity of the gut to maintain the functional network of microbes that keep pathobionts in check.

### The critical window

**Fig. 1:**
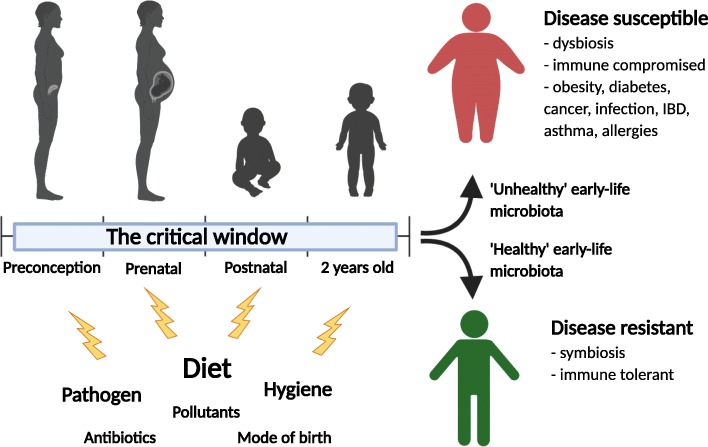
The perinatal period represents a time in development when exogenous factors that affect the microbiome such as antibiotics, diet, hygiene, pathogens, mode of birth, and pollutants can alter immune and physiological programming. The effects of early-life programming may lead to increased disease susceptibility later in life. Created with BioRender.com

The critical window of development is theorized to begin during the preconception period, lasting from conception to up to the first 1000 days of life [5]. It is considered the period during development characterized by greatest phenotypic plasticity, and during which exogenous factors such as diet, antibiotics, mode of birth, and pollutants may lead to long-term physiological and immunological programming [35, 36]. The “fetal programming hypothesis” suggest that maternal nutrition and exogenous factors have long-term metabolic, immune, cardiovascular, and central nervous system effects on their offspring [37–39]. Moreover, the “missing microbe hypothesis” that occurs over-generations is believed to increase disease susceptibility due to suboptimal microbe-host mediated immune development. For instance, the adoption of a low-fiber diet is considered a major player responsible for the intergenerational disappearances of microbes that may promote gut stability and resiliency [40, 41]. Exogenous factors that alter early colonization and succession of microbes in the gut may delay gut maturation and development. Disruptions to microbial networks during this critical window of development are associated with asthma, allergies, diabetes, inflammatory bowel disease, and obesity [35, 42]. It is during this time that the host forms a mutualistic or immune-tolerant relationship with microbes and is thought to alter disease susceptibility (Fig. 1).

### Hygiene

As stated in the UN Millennium Development Goals, the supply of potable water and proper sanitation to control gastroenteritis and malnutrition are important targets across the globe. Intervention trials in these areas, also referred to as WASH (water supply, sanitation, and hygiene), involve the provision of water quality at a public or household level, improved means of excreta disposal, health, and hygiene education, and promotion of handwashing. It has been suggested that EE is a contributor to malnutrition and growth delay in children living in areas with poor sanitation. A systematic review by Gera et al. of trials conducted over the past 10 years in developing countries [43] documented a modest effect of WASH interventions on most anthropometric parameters and studies were of low-quality evidence. Improvement in water quality was associated with a slightly higher weight-for-age *Z*-score (*p* = 0.06). Combined water, sanitation, and hygiene intervention improved height-for-age *Z*-scores (MD 0.22; 95% CI 0.12, 0.32) and decreased the risk of stunting (RR 0.87; 95% CI 0.81, 0.94). Several ongoing trials on these interventions may improve the quality of evidence on infant malnutrition. In a more recent trial of WASH and de-worming in Timor-Leste [44], a trend was observed for a reduction in levels in young children of the fecal EE biomarker, myeloperoxidase; this finding was statistically significant when household water was stored in covered containers.

On the other hand, improved hygiene through regular use of household cleaning products in developed countries has had unintended consequences on infant growth. Despite their widespread use since the late 20th century, antimicrobial cleaning products have not always reduced infection rates of household members, and they release chemicals into the indoor environment [45]. Based on data from full-term infants in the Canadian Healthy Infant Longitudinal Development (CHILD) birth cohort, it was found that infant exposure to frequent cleaning with household disinfectants was associated with altered gut microbial composition at age 3–4 months and risk of being overweight by age 3 [46]. The main attribute of hygiene on malnutrition is immune and gut maturation, where excessive hygiene may hamper symbiont colonization and too little may increase pathogen load.

## The diet-microbe link

**Fig. 2:**
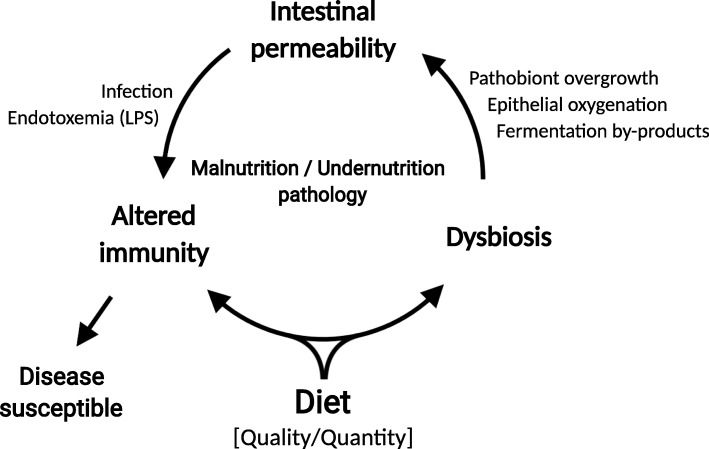
Diet is a major contributor to the pathology of malnutrition and undernutrition. Nutrient quality and quantity can alter host immunity directly and through alteration of gut microbial communities. Dysbiosis adds to the cycle of malnutrition through dietary fermentation by-products, pathobiont overgrowth, and epithelial oxygenation. These conditions alter intestinal permeability, leading to increased pathogen susceptibility and endotoxemia that hampers host immunity and increases disease susceptibility. Created with BioRender.com

As in many cases, there is a constant struggle between cause and effect with respect to microbial dysbiosis and disease outcome in the study of malnutrition and the microbiome. Although the debate to what effect the microbiota contributes to disease remains unclear, it is reasonable to conclude that both gut microbes and environment contribute to the pathogenesis of malnutrition and undernutrition (Fig. 2). Dietary components play a major role in maintaining microbe-host interactions that may promote intestinal health [47]. Therefore, nutrition is an important tool that can be manipulated to restore beneficial microbe-host interactions that enhance intestinal integrity and health.

### Protein

Beginning in the 1950s, protein-energy malnutrition (PEM) became a primary focus associated with stunting and disease susceptibility in children from developing countries [48]. Food shortage is the main culprit of PEM and is estimated to affect 1 in 4 children under the age of 5 worldwide [49, 50]. The prevalence of stunting and wasting, particularly in developing countries, is attributed to low protein-energy intake and increased exposure to enteric infections as a result of poor sanitation [51]. The “protein gap” was considered the primary reason for PEM; however, after much debate, children in developing countries were actually estimated to consume adequate protein, even above recommended levels [48, 52]. The prevalence of stunting could not be explained by protein intake alone and instead has been attributed to intestinal dysfunction and poor energy density from weaning food in developing countries [49, 52]. Unabsorbed proteins can alter intestinal dynamics directly by disrupting gastrointestinal enzymes, receptors, or other activities [53]. Indirectly, protein malabsorption in the small intestine can lead to microbial proteolytic fermentation by-products (H2, CO2, CH4, H2S, short-chain fatty acids (SCFAs), branched chain amino acids (BCAAs), nitrogenous compounds, phenols, and indoles) in the colon with poorly understood health outcomes [54, 55]. Using national level data collected from 180 countries, Ghosh and colleagues concluded that dietary utilizable protein provides a better index of protein inadequacies than total crude protein intake alone [56]. An individual’s protein requirement should be based on protein quality and digestibility, along with the burden of infection and energy deficits of the individual [48, 56]. In addition, microbe-host interactions directed by dietary proteins can provide further insight to assess protein quality and requirements that optimize intestinal health, especially during early development.

Protein-insufficient diet models are used to study the effects of protein malnourishment on intestinal health [17, 57]. Reducing dietary protein in mice from 20 to 5% has drastic consequences on growth stunting, intestinal permeability, and immune function resulting in intestinal inflammation with features similar to EE in humans [17]. Dietary protein provides essential amino acids for protein synthesis, as well as is important for satiety, glucose, and lipid metabolism, blood pressure, bone metabolism, and immune function [53]. Studies show a unique microbial signature specific to protein-deficient diets, distinguished by an increase in Bacteroidetes and Proteobacteria species, and a decrease in *Lactobacillaceae* and *Erysipelotrichaceae* in the gastrointestinal tract [17, 57]. A decrease in *Lactobacillaceae* and *Erysipelotrichaceae* family members are associated with intestinal integrity dysfunction, inflammation, and increased susceptibility to enteric pathogens in mice [58, 59]. Similarly in humans, the population of Bacteroidetes and Proteobacteria species has been shown to be high, while *Lactobacillaceae* species stay low as assessed in a Malawian twin study that evaluated kwashiorkor disease [18]. Early-life protein restriction post-weaning in mice led to temporary glucose intolerance following nutritional recovery but did not increase susceptibility to diet-induced liver steatosis and insulin resistance later in life [60]. A critical window may exist during gestation and suckling prior to weaning that this study did not include and may explain the transient metabolic dysfunction associated from post-weaning dietary protein deficits.

Aside from the microbiota, there is evidence suggesting that maternal protein consumption can impact the metabolism and immune system of their offspring. A high-protein maternal diet was able to stimulate hypothalamic MAPK insulin signaling pathways that are involved in controlling energy and nutrient homeostasis [61]. A diet low in protein was determined to activate the liver and hypothalamus hedgehog-signaling pathway that is positively correlated with liver disease (e.g., NAFLD) and hepatic repair mechanisms [61]. A low-protein diet during pregnancy and lactation leads to increased inflammatory status as determined by the expression of microRNAs (miRNAs), and levels of inflammatory IL-6 and TNF-α markers in mice [37]. miRNAs are shown to be involved in the pathogenesis of metabolic disorders by regulating insulin signaling, immune-mediated inflammation, adipokine expression, adipogenesis, lipid metabolism, and food intake [62]. In pigs, a low-protein diet increased cortisol and decreased protein levels in sows, and led to high cortisol, low IgA, and increased mortality in suckling piglets [63]. The high-protein diet decreased systemic immunoglobulin (IgA, IgG, and IgM) and increased systemic CD4^+^ lymphocytes and CD4^+^/CD8^+^ ratio in weaned piglets indicating altered immune function. An optimal quantity of dietary protein may exist because both low- and high-protein diets increased IL-6 in the blood of piglets challenged with lipopolysaccharide (LPS) compared to normal protein feeding [63]. Maternal high-protein (40% of diet) diets during pregnancy, but not lactation, alter hepatic gene expression in adult mouse offspring, suggesting permanent imprinting on metabolic function [64]. A small number of genes persisted in adulthood, including those involved in liver regulation, damage, and metabolic dysfunction (*Hifla* and *Hnf4a*), and genes that regulate liver DNA methylation (*Mecp2* and *Sin3a*) that explain the long-term imprinting effects of a prenatal high-protein diet [64]. Moreover, glucagon-like protein-1 (GLP1) was higher in high-protein offspring (protein induces satiety) and was associated with reduced insulin sensitivity as determined by high insulin serum levels and increased resistin and IL-6 expression in brown adipose tissue in rats [65]. Rat offspring from dams given a high-protein diet during gestation had a 41.2% increase in adiposity correlating with a significantly increased phosphoenolpyruvate carboxykinase (PEPCK; a gluconeogenesis enzyme) and altered insulin signaling compared to normal protein maternal diets [66]. A high-protein diet during gestation and lactation is shown to increase plasma insulin when rat pups are weaned on a normal protein diet; however, weight gain remained the same between groups [67]. This research indicates that maternal and infant protein consumption may contribute to metabolic programming lasting into adulthood. Yet, the discrepancy between some studies linking high-maternal-protein diets to offspring weight gain may indicate a role of the gut microbiota and requires further investigation.

The amount of protein consumed early in life may play an important role in programing metabolic function. Although the mechanism of programming is poorly defined, the effects of dietary protein may have a microbial component. The “early protein hypothesis” suggests that the consumption of a high-protein diet with sufficient energy, such as the typical infant formula, increases infant weight gain, adipogenic activity, and obesity risk later in life [68]. During the first 6 months of life, protein intake has been shown to be 66–70% higher in formula-fed (FF) than in breastfed (BF) infants leading to greater weight gains in infants from 3 to 9 months [69]. This is partly due to the fact that infant formula contains higher concentrations of protein than human breast milk. Protein content of human breast milk ranges from 1.4–1.6 g/100 mL to 0.8–1.0 g/100 mL after 4 months of lactation, whereas infant formula ranges from 2 to 3.5 g/100 mL, with a higher protein formula given to low birth weight or preterm infants [70]. In a multicenter, double-blind, randomized clinical trial, high-protein FF infants (6 years old) had a significantly higher body mass index (BMI) and were twice as likely to develop obesity compared to the low-protein FF infants [71]. Limiting protein intake may help reduce the effects of long-term metabolic programming; however, the events leading to this observation and cause are still poorly understood. A targeted serum metabolome analysis revealed BF infants demonstrated increased fat metabolism, whereas FF infants had a metabolite profile indicative of protein metabolism [72]. The greatest difference was determined at 2 months (baseline), 4 months, and 6 months of age, and remained different when “carbohydrate-rich” complementary foods were added to the diet. Fecal microbiome structure and metabolomic profile mirrored changes associated with serum metabolite profiles [72, 73]. As expected, protein-rich infant formula induced microbial end-products typical of peptide and amino acid fermentation such as branched chain fatty acids (BCFAs; isobutyrate and isovalerate) and phenylacetate [73]. The contribution of the microbiota to the relationship between early-life protein and long-term metabolic outcomes has not been explored in a controlled system; however, given the evidence linking early-life microbial disruptions with long-term disease risk supports a role of protein-microbe interactions.

Excessive protein intake can lead to protein fermentation products known to disrupt gastrointestinal tract function and contribute to the pathogenesis of irritable bowel syndrome and ulcerative colitis, with a distinguishing malodorous flatus [54]. Plant-based diets from developing countries such as bean and cowpea flour are primarily poor in protein quality and reduced linear growth compared to fish protein-rich diets [74]. Maize and cassava are other popular protein sources in developing countries that are poor sources of tryptophan and lysine [75]. Microbiota-derived tryptophan metabolites are instrumental in maintaining intestinal homeostasis [76]. In fact, tryptophan is considered the limiting amino acid in infant formula (~ 250 mg/L) due to low bioavailability from bovine milk protein. Formula would require twice as much tryptophan to reach the bioavailable levels similar to that of breast milk (~ 200–400 mg/L) [72]. Processes that disrupt microbial activity and reduce tryptophan metabolite capacity can lead to increased digestive dysfunction and pathogen susceptibility [77, 78]. In mice, dietary amino acid metabolism has been mechanistically linked to angiotensin I-converting enzyme 2 (Ace2) deficiency by altering microbial gut ecology and antimicrobial peptide expression, increasing infection susceptibility [79]. Bioavailability of certain amino acids may play an instrumental role in diet-induced dysfunctions leading to PEM. Protein source and digestibility can impact microbe-host interactions in the gut, perpetuating malnutrition by decreasing intestinal integrity and function [47]. More research is needed to determine to what extent the microbiota and host intestinal activities are programmed through maternal exposure to the amount and source of proteins.

### Fats

A universal deficiency that occurs in malnutrition, both energy and PEM, is a less than sufficient intake of fat and/or essential polyunsaturated fatty acids. Although the gut microbiota is recognized as an important pathophysiologic factor in the development and sustainment of malnutrition, there are few studies that have specifically examined their impact in relation to dietary fat. It is, however, generally accepted that the bidirectional interactions between the microbiome, fat availability, and gastrointestinal function contribute to a vicious circle, further impairing health outcome in malnutrition [80].

Although gut microbes have been implicated in regulating fat absorption and metabolism [81], a comprehensive and mechanistic approach to understanding the pathways is still needed. In a healthy infant, > 95% of fats are absorbed before entering the large intestine. Malnutrition, especially in infants/children results in a generalized disturbance of the small intestine structure (shortened, blunted villi, and increased crypt depth) and function (disturbances in permeability and absorption) resulting in fat malabsorption [80]. This results in diarrhea, weight loss, other nutritional deficiencies, and gut dysbiosis [80]. The accompanying intestinal and systemic inflammation are suggested to contribute to these pathological changes [80, 82]. Additionally, the microbiota plays a major role in bile acid deconjugation [83]. Altered microbial deconjugation of bile acids would further impair absorption of fat, cause bile salt injury to the colon, and interfere with digestive enzymes and nutrient transporters [80].

In the colon, microbial perturbations can indirectly alter energy balance via changes in the levels of anorectic hormones (peptide YY (PYY) and GLP-1) and effects on energy (both lipid and glucose) metabolism [84]. The gut microbiota has been demonstrated to stimulate hepatic triglyceride production (lipogenesis) by activating the transcription factors, carbohydrate response element binding protein (ChREBP), and sterol response element binding protein (SREBP) [85]. Recently, a critical role of the, complex but less diverse, small intestine microbiota as a regulator of fat digestion and absorption has been identified [86, 87]. These microbes appear to be essential for host adaptation to dietary lipid changes by regulating gut epithelial processes involved in their digestion and absorption. It is hypothesized that this occurs via systemic control of enteroendocrine signaling and an effect on erythrocytes fatty acid transport [87]. As fat is the major energy source for infants and children, an inadequate supply can lead to long-term health consequences to the infant/child through the impact on inadequate energy reserve, epigenetic changes in cells critical for growth, long-term defects in the small intestine and immune maturation, and recurrent bouts of infection [82]. Changes to the microbiome early in life could also have lasting effects on the ability to adapt to changes in fat intake later in life, thereby contributing to metabolic disorders [87].

A supply of both the n-6 and n-3 PUFA is essential for growth and intestinal, neurocognitive, visual, and immune development [88, 89]. Among the n-3 PUFA, eicosapentaenoic acid (EPA; C20:5) and docosahexaenoic acid (DHA, C22:6) are the two main bioactive forms in humans and for the n-6, arachidonic acid (Ara, C20:4). An n-3 PUFA deficiency, especially during intrauterine and early life, is associated with impaired psychomotor development, and issues with attention, cognition, and visual acuity [90]. An insufficient supply of PUFA alters systemic and intestinal immune development, which occurs postnatally within the first 3 years of life [89]. However, the development of the immune system is closely tied with that of the microbiome [85]. Despite this knowledge, the impact of essential fatty acid deficiency on the microbiome has not been well studied.

Studies have shown that different types of dietary fat, including saturated fatty acids (SFAs), monounsaturated fatty acids (MUFAs), and PUFAs, and their abundance in the diet, could change gut microbiota composition [91]. There is growing evidence for a potential role of a disrupted microbiota in metabolic disorders induced by n-3 PUFA deficiency. Studies have shown that dietary n-3 PUFAs can modify the intestinal microbiota composition by increasing the number of bifidobacteria that decrease gut permeability and decreasing the number of Enterobacteria that increase intestinal permeability [92]. Animal studies have reported that supplementation of n-3 PUFA in young rodents can restore the disturbed gut microbiota composition of maternally separated female rats [93] or the disrupted function of the microbiome in male pups [90]. The position of the DHA or ARA in milk triacylglycerols has also been demonstrated to modify the intestinal bacterial population of the suckling rodent [94]. The effect of these fats on the microbiota is best explained by indirect mechanisms. This is particularly evident in a study that demonstrated differences in microbial composition in response to changes in the parenteral fatty acid formulations [95].

The impact of PUFA on the gut microbiota is less well defined. The few studies completed in adults showed some common changes in the gut microbiota after n-3 PUFA supplementation. In particular, a decrease in *Faecalibacterium*, often associated with an increase in the Bacteroidetes and butyrate-producing bacteria [92]. Although the literature on this topic is discordant, n-3 PUFAs are generally associated with anti-inflammatory effects, in comparison with the omega-6 PUFAs that are linked to pro-inflammatory effects, due to the different downstream lipid metabolites [92]. Supplementation with n-3 PUFAs can exert a positive action by reverting the microbiota composition in adult inflammatory diseases and increase the production of anti-inflammatory compounds, like short-chain fatty acids [92]. In addition, accumulating evidence in animal model studies indicates that the interplay between gut microbiota, n-3 PUFA, and immunity helps to maintain intestinal integrity and influences the gut–brain axis, acting through shifts in the microbial gut network [92]. Infant trials have demonstrated that supplementation with PUFA results in greater bacterial diversity combined with lower abundance of some pathogenic bacteria, such as *Streptococcus, Clostridium*, and some genera of the *Enterobacteriaceae* family, such as *Escherichia*, *Klebsiella, Serratia*, and *Citrobacter*, suggesting that n-3 PUFAs favor the butyrate-producing bacterial genera [96]. Identifying direct and indirect attributes of dietary fats on microbial networks and immune function will help elucidate the contribution of dietary fat type and quantity on malnutrition status.

### Carbohydrates

The main contribution of carbohydrate in human nutrition is to provide glucose for supporting the high energy demands of the brain and muscular system [97, 98]. Experts recommend an intake of 150 g/day in adults to support muscle and brain physiology, but no minimum requirement has been established [99, 100]. Humans have thrived on diets containing various amounts and types of carbohydrates. However, the excessive consumption and reliance on poor-quality carbohydrates is thought to have contributed to the increased prevalence of chronic diseases [100]. Although difficult to define, the nutritional quality of a carbohydrate can be described by their digestibility and activity on the microbiota.

Unabsorbed carbohydrates are fermented by gut bacteria into lactic acid, succinate, SCFAs (acetate, propionate, and butyrate), and hydrogen, methane, and carbon dioxide gases [101]. SCFAs are associated with improved intestinal and metabolic health; however, it may also contribute to a variety of gastrointestinal symptoms including abdominal cramps, bloating, flatulence, and diarrhea [102]. Carbohydrate malabsorption is frequently reported in severely malnourished children; however, to what extent it contributes to the pathologies surrounding malnutrition remains unclear [103]. Population-based studies that followed the introduction of refined carbohydrates to the diet have shown an increased prevalence of atherosclerotic disease, coronary heart disease, and diabetes [104, 105]. The risks associated with diets high in simple sugars (e.g., sucrose, glucose, fructose) is considered a result of impaired intestinal permeability due increased LPS endotoxemia and loss of luminal SCFAs [106, 107]. Although a strong link exists between a high simple sugar diet and poor health outcomes, research suggests that dietary fiber and resistant starches may be more important than quantity and glycemic load of simple carbohydrates [108].

Dietary fiber and resistant starch are complex polysaccharides that support homeostasis through their effects on the intestinal mobility and microbiota. The recommended adequate intake of dietary fiber is 14 g/1000 kcal, equating to approximately 28–36 g/day in human adults, a number that many experts believe should be closer to 50 g/day [109, 110]. The bulking properties of dietary fiber are well known to positively influence gastric emptying, fecal frequency, and satiety [111]. The production of SCFAs, specially butyrate, provides direct energy to gut epithelial cells and promotes intestinal homeostasis by maintaining a hypoxic environment, mucus production, and antimicrobial peptide secretion [112–116]. Commonly known as prebiotics or microbial-accessible carbohydrates (MACs), fibers and starches that encourage SCFA production also promote microbial diversity and resiliency. In mouse models, these attributes have consistently supported the positive effect of MACs on intestinal integrity [117, 118]. A MAC-deficient diet is hypothesized to have contributed to gut microbe extinctions across generations, leading to reduced diversity and SCFA production, and dysbiosis associated to disease later in life [5, 41]. The type and quantity of MACs directly alters microbial gut networks and fermentation products, such as SCFAs, wherein effects on host physiology and health have been exhaustively reviewed [109, 118, 119]. To what extent dietary MACs shape early-life development and disease risk remains unknown and requires well-controlled studies comparing MAC type and introduction to the diet during the critical window of development.

### Iron

Iron deficiency (ID) is the most common nutritional deficiency in the world and is highly prevalent in both developed and developing nations. The sub-populations most at risk of ID, irrespective of geographical location, are pregnant women and young children [120]. The world health organization (WHO) estimates that 38% of all pregnant women (~ 496 million worldwide) and 43% of children (~ 273 million worldwide) are anemic [121], of which more than half are attributed to ID [120]. Early childhood is characterized by increased demands for iron, which is needed to support tissue and organ development as well as blood volume expansion. Consequently, reduced iron availability (due to inadequate intake or absorption, chronic inflammation, or insufficient iron accretion and storage in gestation) during this critical period can lead to suboptimal growth, with potential consequences for long-term health. Indeed, epidemiological studies show ID in childhood is associated long-lasting deficits in cognitive ability, memory, and executive function [122–125]. Studies in rats show that maternal iron restriction in pregnancy and consequent ID in offspring affects growth and developmental trajectories, with lasting effects on behavior and cognition [126], and cardiovascular and metabolic health [127–131].

The mechanisms by which ID impacts offspring development are complex and likely depend on the organ system in question, but pathophysiological processes including hypoxia [132], altered energy metabolism, reactive oxygen species generation [133], epigenetic changes [134, 135], and altered neurotransmitter and hormonal profiles [136–138] are implicated. More recently, the profound modulatory effects of iron availability on gut microbiome composition and function have been demonstrated [139, 140], with implications for metabolic substrate production [139, 140] and risk of infection by enteropathogens in young children with ID. Indeed, just as iron is essential for eukaryotic cell function, most bacterial species depend on iron for survival and pathogenicity [141–143], and as such, availability of iron may play an important role in microbiome niche selection.

In young children, ID anemia was associated with increased enterobacteria and *Veillonellaceae*, reduced *Coriobacteriaceae*, and reduced the bifidobacteria/enterobacteria ratio [144]. A study by McLorry et al. also showed that anemia in boys and girls was associated with lower levels of butyrate-producing bacteria (*Butyricicoccus* in females, *Coproroccus* and *Roseburia* in males) [145]. In general, these reports are consistent with in vitro studies using a continuous gut fermentation model inoculated with child gut microbiota [139, 140]. In these experiments, Dostal et al. reported that low iron conditions reduced butyrate-producing bacteria (e.g., *Roseburia* spp., *Eubacteria rectale*, *Clostridium* cluster IV members, and *Bacterioides* spp.) and reduced butyrate concentrations and other SCFA (acetate and propionate) [139, 140], and these effects could be reversed with provision of iron [139]. The authors also recapitulated these trends in vivo in weaning rats [146].

However, the influence of iron status on microbial composition and function in children is complicated by several population factors, including enteropathogen exposure (e.g., high vs. low pathogen burden [147]), anemia identification with and without iron deficiency, dietary fortification regimens, and use of iron supplements. The latter is particularly notable, because iron supplementation is routinely implemented in populations at risk of ID, including young children [148]. In excess, unabsorbed iron remains in the gastrointestinal tract, where it may provide a labile iron pool for bacteria. Notably, several species of Proteobacteria (e.g., *Enterobateriaceae* such as *Escherichia coli* and *Salmonella*) and Firmicutes (Bacillus) require iron to colonize and persist in the intestine [149]. In an iron-rich colonic environment, such as that produced by excess iron supplementation, Proteobacteria may thrive at the expense of other gut bacteria [150, 151]. Consistent with this idea, studies show that supplementation with iron supplements and iron-rich micronutrient powders can increase diarrhea risk, which may be mediated by increased abundance of Enterobacteria (including *E. coli*) and reduced abundance of beneficial commensal bacteria (e.g., bifidobacteria, lactobacilli) [152–154]. The risks and benefits of iron supplements and micronutrient powders in children, particularly in low- and middle-income countries, is an active area of research, and several excellent reviews on the topic are currently available [148, 155].

It is interesting that ID anemia, which is presumably associated with reduced iron availability in the gut, is associated with increased abundance of Enterobacteria (as discussed above), given the iron requirements for colonization and virulence. However, many members of this group are known to use siderophores, high-affinity compounds that bind and sequester iron, and other specialized iron transport systems which enable these bacteria to effectively compete for iron when availability is scarce [141]. In their study, Dostal et al. showed that the abundance of *Enterobacteriaceae* increased with low iron, but decreased with provision of FeSO_4_ [146]; the latter finding may reflect a loss of this competitive advantage with increasing iron availability, and the reduced growth may be secondary to growth promotion of other bacterial groups (e.g., *Allobaculum* spp.). Notwithstanding, these studies highlight a complex but important interrelationship between the microbiota in childhood and iron status, and highlight a myriad of influences on this relationship, including use of iron supplements and their formulations, population characteristics, presence of other nutritional deficiencies, or excesses among others.

### Vitamin D

Vitamin D is a fat-soluble vitamin with many molecular forms. A paper by Mokhtar and colleagues on vitamin D status and growth in Ecuadorian children is an important contribution [156]. Mean serum 25(OH)D levels did not differ between underweight (WAZ < − 1.0) and non-underweight children, although underweight children were twice more likely to have serum vitamin D levels < 42.5 nmol/L. In order for vitamin D to elicit a physiological response, it must be converted in the liver and kidney to its active form, calcitriol (1,25(OH)2D3), but it must also bind with the nuclear vitamin D receptor (VDR) [157]. VDR is highly expressed in the proximal colon and acts as a transcription factor responsible over 1000 genes, including the defensins, cathelicidin, claudins, TLR2, zonulin occludens, and NOD2 [158, 159]. These proteins are major drivers of inflammation; they help with the maintenance of the host barrier and promote tolerance to cytokines and the gut microbiota. Given the role of VDR and downstream regulators of microbe-host interactions, it is worthwhile considering the evidence on interactions between vitamin D and gut microbiota in early life, especially in view of the important role of infant gut microbiota in child weight gain [160].

A recent systematic review provides up-to-date information regarding vitamin D and the gut microbiota in animal, cell, and human studies [161]. In animal studies which typically use VDR KO (knock-out) mice, microbial “dysbiosis” has been reported, characterized by a greater abundance of Proteobacteria, *Bacteroides* spp. and *Clostridium* spp. [162, 163]. Few studies have been published in human adults. In a cross-sectional analysis of 150 healthy adults, Luthold and colleagues found higher levels of serum 25(OH)D to be associated with greater abundance of *Bifidobacterium* and *Prevotella* in gut microbiota but a depletion in *Haemophilus* and *Veillonella* [164]. Stool samples and biopsies obtained from the gastrointestinal tract of 15 healthy adults taken before and after supplementation with high doses of vitamin D3 showed higher microbial richness and enrichment with Proteobacteria in biopsies; no significant changes to the microbial composition in stool samples [165]. Finally, human adults who are deemed to have an “insufficient” serum vitamin D level have two times greater odds of acquiring *Clostridium difficile* infections at hospitalization, compared to patients who are vitamin D deficient [166].

The evidence in human infants is even more sparse. The KOALA birth cohort study examining both maternal and infant vitamin D supplementation found that infant direct supplementation (i.e., via liquid drops) did not alter gut microbial composition at 1 month after birth [167]. However, indirect vitamin D exposure, specifically maternal prenatal multivitamin supplementation, was correlated with greater abundance of *Bacteroides fragilis* and reduced presence of *Bifidobacterium* spp. and *C. difficile.* In the Vitamin D Antenatal Asthma Reduction (VDAART) Trial, mothers of infants received either 4000 IU vitamin D plus prenatal vitamins or 400 IU vitamin D plus prenatal vitamins during the first trimester of pregnancy [168]. There were no observed differences between the diversity of microbiota in the two exposure groups at 3–6 months of age. However, enrichment with *Lachnospiraceae* and *Lachnobacterium* and depletion of *Lactococcus* were observed in infants with higher vitamin D levels in cord blood.

Maintaining adequate vitamin D levels is crucial for vitamin D-dependent immunity and physiology, which is particularly important during growth and development. Increasing vitamin D status through nutrition, supplementation, or sun exposure will modulate the gastrointestinal microbiota by supporting the gut environment and normalizing autoimmune responses [169]. Although vitamin D can be treated with supplementation and increased sunlight exposure, genetic factors, malabsorption (i.e., EE), and underlying conditions, such as cystic fibrosis, celiac disease, and IBD, can lead to deficiencies [170]. Future studies should focus to delineate between host physiological and immune changes surrounding vitamin D status that may alter the gastrointestinal environment and the succession of microbes to children from vitamin D-deprived mothers at birth.

### Vitamin B12

Vitamin B12, also known as cobalamin, is a water soluble cobalt-containing vitamin that is exclusively synthesized by bacteria and archaea [171]. In humans, it is essential for DNA synthesis, cellular energy production, red blood cell maturation, and myelin synthesis of neural networks. Inadequate absorption of cobalamin from diet leads to anemia and neurologic dysfunction [172]. In malnourished children, vitamin B12 deficiency is more common than folate and iron deficiency and can lead to long-term brain damage [173, 174]. Insufficient vitamin B12 status has been shown to lower cognitive development and performance [175], cause growth and motor retardation [176], and decrease bone mass in children [177].

Many gut bacteria encode for multiple vitamin B12 transporters, while less than 25% are predicted to synthesize it [178]. Because endogenous bacterial production of vitamin B12 occurs in the colon, it is unlikely to significantly contribute to host B12 status. Enterohepatic circulation and microbial remodeling contribute to a pool of at least eight distinct vitamin B12-derivatives (corrinoids) identified in human feces [179]. The presence of multiple corrinoids, corrinoid microbial transporters, and corrinoid-dependent enzymes suggest competition exists between gut bacteria. Research has focused to identify the ability of vitamin B12 to modulate gut microbial ecology and how these interactions influence disease status [180].

Vitamin B12 derivatives are involved in syntrophic microbe-microbe interactions in the gut that help maintain symbiosis and intestinal health [180]. For instance, in vitro co-culture studies suggest *Akkermansia muciniphila*, a mucus-degrading microbe associated with a lean phenotype and enhanced barrier function, may rely on *Eubacterium hallii* to provide them with corrinoids [181]. Competition between gut bacteria for corrinoids occurs when resources are limited or when an increased demand is required [182]. Research has shown that commensal *Bacteroides thetaiotaomicron* is able to outcompete enterohemorrhagic *E. coli* (EHEC) for vitamin B12 via cell surface-exposed lipoproteins [183]. Furthermore, mutant *B. thetaiotaomicron* lacking B12 transporters lose their ability to inhibit EHEC virulence factors [184]. In excess through supplementation, vitamin B12 availability may increase the risk of enteric pathogens in the gut. This is especially important in regions of poor socioeconomically status, where risk of enteric infection is high. Deficiencies treated with supplements in excess can help to restore vitamin B12 status but may also support enteric pathogen fitness contributing to EE and worsening malnutrition status. Future research should focus on the effects of dietary vitamin B12 on microbial gut ecology and intestinal health.

## Conclusion

Controlling the microbiota with targeted dietary interventions, particularly in cases of malnutrition, may prove to be a vital therapeutic option in preventative healthcare. The mounting concerns over early-life nutrition have pointed towards the microbiota and disruptive gut function as a causal factor towards disease susceptibility later in life; however, the direct cause and effect are difficult to tease out. Future nutritional research will require well-controlled studies of a nutrient on its own and in combination with other components that stress and protect the gut. Using an omics and an extensive host phenotyping approach will help unravel the complex interactions between the gut microbiota and health of malnourished children.

## Abbreviations

DOHaD: Developmental origins of health and disease
IBD: Irritable bowel disease
EE: Environmental enteropathy
WASH: Water supply, sanitation and hygiene
CHILD: Canadian Healthy Infant Longitudinal Development
PEM: Protein-energy malnutrition
SCFAs: Short-chain fatty acids
BCAAs: Branched chain amino acids
MAPK: Mitogen-activated protein kinase
NAFLD: Nonalcoholic fatty liver disease
miRNAs: MicroRNAs
LPS: Lipopolysaccharide
PYY: Peptide YY
GLP1: Glucagon-like protein-1
PEPCK: Phosphoenolpyruvate carboxykinase
FF: Formula-fed
BF: Breastfed
BCFA: Branched chain fatty acids
Ace2: Angiotensin I converting enzyme 2
ChREBP: Carbohydrate response element binding protein
SREBP: Sterol response element binding protein
PUFA: Polyunsaturated fatty acid
EPA: Eicosapentaenoic acid
DHA: Docosahexaenoic acid
SFAs: Saturated fatty acids
MUFA: Monounsaturated fatty acids
ARA: Arachidonic acid
MACs: Microbial-accessible carbohydrates
ID: Iron deficiency
WHO: World Health Organization
VDR: Vitamin D receptor
KO: Knock-out
EHEC: Enterohemorrhagic *Escherichia coli*
